# A genomic approach to investigate developmental cell death in woody tissues of *Populus *trees

**DOI:** 10.1186/gb-2005-6-4-r34

**Published:** 2005-03-22

**Authors:** Charleen Moreau, Nikolay Aksenov, Maribel García Lorenzo, Bo Segerman, Christiane Funk, Peter Nilsson, Stefan Jansson, Hannele Tuominen

**Affiliations:** 1 Department of Plant Physiology, Umeå Plant Science Centre, Umeå University, SE-901 87 Umeå, Sweden; 2 Department of Biochemistry, Umeå University, SE-901 87 Umeå, Sweden; 3 Department of Biotechnology, KTH - Royal Institute of Technology, AlbaNova University Center, SE-10691, Stockholm, Sweden

## Abstract

**Background:**

Poplar (*Populus *sp.) has emerged as the main model system for molecular and genetic studies of forest trees. A *Populus *expressed sequence tag (EST) database (POPULUSDB) was previously created from 19 cDNA libraries each originating from different *Populus *tree tissues, and opened to the public in September 2004. We used this dataset for *in silico *transcript profiling of a particular process in the woody tissues of the *Populus *stem: the programmed death of xylem fibers.

**Results:**

One EST library in POPULUSDB originates from woody tissues of the *Populus *stem where xylem fibers undergo cell death. Analysis of EST abundances and library distribution within the POPULUSDB revealed a large number of previously uncharacterized transcripts that were unique in this library and possibly related to the death of xylem fibers. The *in silico *analysis was complemented by a microarray analysis utilizing a novel *Populus *cDNA array with a unigene set of 25,000 sequences.

**Conclusions:**

*In silico *analysis, combined with the microarray analysis, revealed the usefulness of non-normalized EST libraries in elucidating transcriptional regulation of previously uncharacterized physiological processes. The data suggested the involvement of two novel extracellular serine proteases, nodulin-like proteins and an *Arabidopsis thaliana OPEN STOMATA 1 *(*AtOST1*) homolog in signaling fiber-cell death, as well as mechanisms responsible for hormonal control, nutrient remobilization, regulation of vacuolar integrity and autolysis of the dying fibers.

## Abstract 2

A *Populus *EST dataset was used for *in silico *transcript profiling of the programmed death of the xylem fibres in woody tissues of *Populus *stem. The analysis suggests the involvement of two novel extracellular serine proteases, nodulin-like proteins and an *AtOST1 *(*Arabidopsis thaliana OPEN STOMATA 1*) homolog in signaling fiber-cell death.

## Background

The woody tissues of angiosperm trees, the xylem fibers and vessels, are formed from the lateral meristem of the stem, the vascular cambium. In contrast to vessel elements, which differentiate very rapidly close to the vascular cambium, fiber differentiation is a relatively slow process involving initial expansion of the cells in both the radial and longitudinal dimensions, followed by extensive synthesis of the secondary cell walls. The final phase in maturation of both vessel elements and fibers is cell death and autolysis of the cell contents.

Xylem-cell death involves a range of morphological and nuclear changes in a strictly spatially and temporally coordinated and programmed fashion [1,2]. The programmed cell death (PCD) of xylem has been analyzed in detail in an *in vitro *system of *Zinnia elegans*, in which mesophyll cells of *Zinnia *transdifferentiate into xylem vessels commonly called as tracheary elements in a semi-synchronized manner [3]. In *Zinnia *cells, irreversible differentiation into tracheary elements is marked by the accumulation of hydrolytic enzymes in the vacuole and deposition of the secondary cell walls, followed by tonoplast disruption, release of the vacuolar proteases and nucleases into the cytoplasm, and finally the autolytic loss of cell contents [2,4]. Several different types of proteases have been detected in *Zinnia *[5,6], and an S1-type nuclease, capable of hydrolyzing both DNA and RNA, seems to control nuclear DNA degradation in the *Zinnia *tracheary elements [7]. Even though the chain of events during tracheary element PCD is well characterized in the *Zinnia *system, very little is known about the regulation of this process in intact plants. In addition, the *Zinnia *system has not allowed analysis of the different cell types of the xylem, such as the fibers.

Programmed cell death also occurs in plants in response to external factors, such as avirulent pathogens, giving rise to the so-called hypersensitive response (HR) and in response to shortening daylength - manifested in the senescence of leaves. HR cell death is usually fast and it shares certain features with the apoptotic death of animal cells, such as nuclear shrinkage and fragmentation of DNA into oligonucleosomal multiples of 180-bp fragments [8]. Senescence-induced cell death is a much slower process, involving nuclear degradation, DNA fragmentation and thorough proteolytic degradation of the cellular contents and controlled remobilization of the nutrients [9]. The death of the xylem elements is different from HR and senescence-related PCD in that the organellar structure remains intact until vacuolar collapse and the oligonucleosomal DNA fragmentation does not precede cell death [1]. Whether these processes are related at the molecular level is unknown, but the differences in temporal and spatial regulation, and in cellular morphology, suggest that there are significant differences not only in the early regulation, but also in the execution of the various plant PCD processes.

The genus *Populus *has emerged as the main model system for trees, because of its amenability for genomic and molecular analyses [10]. *Populus *is also suitable for analysis of xylem development [11,12]. A *Populus *expressed sequence tag (EST) database (POPULUSDB) was created from 19 different cDNA libraries [13]. The database consists of 102,019 ESTs, assembled into a unigene set of 11,885 clusters and 12,759 non-clustered singletons corresponding altogether to 24,644 unique sequences or transcripts [14]. The great diversity of the tissue types giving rise to the different cDNA libraries enables digital analysis of gene expression by comparison of the EST frequencies in the different libraries. One of the libraries was produced from *Populus *woody tissues composed of xylem fibers undergoing cell death. In this work, we studied gene expression in the process of fiber death by *in silico *analysis of this 'fiber death library' and by a microarray analysis with a novel *Populus *25K cDNA microarray. In addition to its economic importance as one of the processes that regulate wood quality, fiber-cell death is an interesting biological process that as yet is poorly understood. Our analysis identified several novel candidate regulatory genes for xylem PCD.

## Results and discussion

### The unique characteristics of fiber-cell death in *Populus *wood

**Figure 1:**
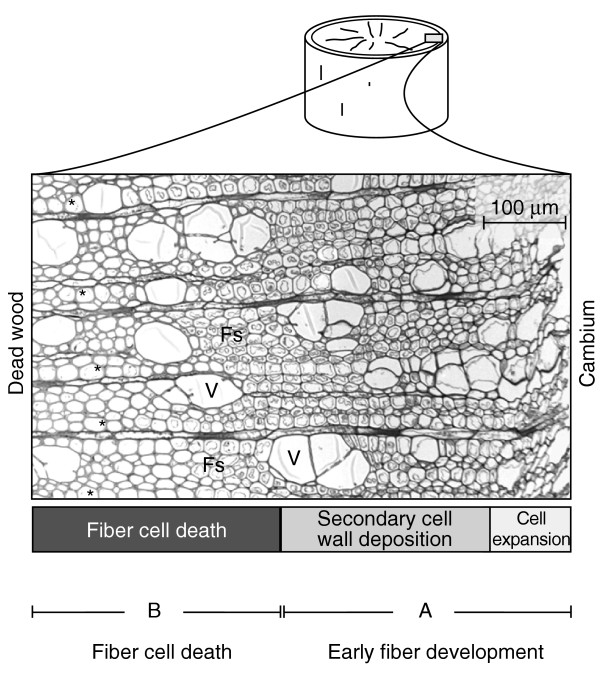
Sampling of xylem tissues. A transverse section from the base of the stem showing xylem tissues sampled from a *Populus *tree for the microarray and the RT-PCR analysis. The bark was peeled off resulting in a fracture in the cambial zone. For RT-PCR analysis, the different xylem tissues were successively scraped from the surface of the exposed stem to the border with the dead wood. For microarray analysis, the tissues were pooled into two samples: A (early fiber development) and B (fiber-cell death). The fiber-cell death sample corresponded closely to the tissues collected for construction of the fiber death cDNA library [13]. V, dead vessel; Fs, developing fibers. Note that the development of vessels is completed within the region of cell expansion, and that the fibers develop at a much slower pace, visualized by the gradual loss of the cytoplasm of the fibers. The asterisks denote fibers close to the moment of death with barely detectable cytoplasm.

An analysis of the fiber death cDNA library in the POPULUSDB was undertaken to characterize specific molecular events in *Populus *xylem fibers approaching cell death. The fiber death library was constructed from xylem tissues in which the fibers had passed the developmental phases of cell expansion and bulk secondary cell wall deposition, and were approaching cell death (see [13], and corresponding to zone B in Figure 1). Cell death of the fibers is marked by gradual disappearance of the cytoplasm and finally by complete autolysis of the cells when no cytoplasm can be discerned within the cells (Figure 1). Differentiation of xylem vessels differs from that of fibers in that it is much faster, occurring usually within a distance of 100-150 μm from the cambium. Development of the vessels is difficult to study *in vivo *not only because it is so fast, but also because it takes place in the midst of xylem fibers that are still finishing cell expansion and initiating secondary cell wall deposition. To avoid mixing different processes of xylem development, we decided in this analysis to exclude woody tissues containing differentiating xylem vessels and to focus purely on the late maturation events of xylem fibers.

**Figure 2:**
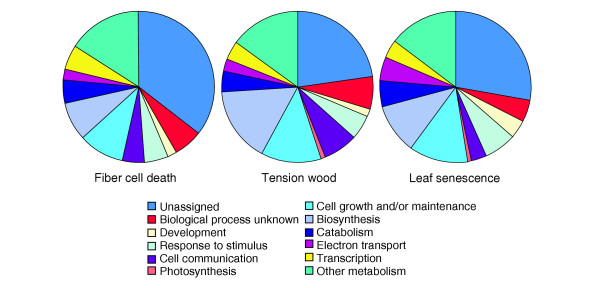
Assignment of gene ontologies to ESTs in the cDNA libraries representing fiber death, tension wood and leaf senescence in the POPULUSDB.

To obtain a broad picture of cell death in xylem fibers, we compared the relative distributions of ESTs with different gene ontology assignations in three cDNA libraries of POPULUSDB: the fiber death library, the tension wood library derived from tension wood-forming xylem, and the leaf senescence library [13]. The leaf senescence library was chosen as it represents another PCD process in plants, and the tension wood library because it represents tissues where fiber death is inhibited but is otherwise comparable to the fiber death library. Tension wood is formed in an asymmetric manner in gravistimulated stems of angiosperm trees. As a part of this process the fibers show delayed cell death due to production of a cellulose-rich layer, the so-called G-layer, inside the secondary cell walls. Remarkably, the fiber death library showed a higher proportion of ESTs (36%) that could not be assigned to any gene ontology term, compared to the tension wood library (23%) and the leaf senescence library (27%) (Figure 2). In addition, 6%, 7% and 5% of the ESTs in the fiber death, tension wood and leaf senescence libraries, respectively, represented unknown biological processes. These figures indicate that unique and poorly characterized physiological processes may occur in xylem fibers undergoing cell death. The two cell-death libraries, the fiber death and the leaf senescence, were similar in the sense that they had fewer clones related to biosynthesis and cell communication, and a larger number of clones related to catabolism than the tension wood library (Figure 2). However, the leaf senescence library had higher frequencies of clones in the categories of electron transport and development than the other two libraries (Figure 2). Altogether, the analysis demonstrated that the distributions of biological processes during fiber-cell death are fairly similar to those during both tension wood formation and leaf senescence. Common features shared by the fiber death and leaf senescence libraries suggest similarities between fiber-cell death and senescence-related PCD. However, fiber-cell death is also expected to involve metabolic and regulatory pathways that have not yet been characterized, based on the high proportion of ESTs with unknown gene ontology or unknown function in the fiber death library.

### The most abundant transcripts during fiber-cell death

**Figure 3:**
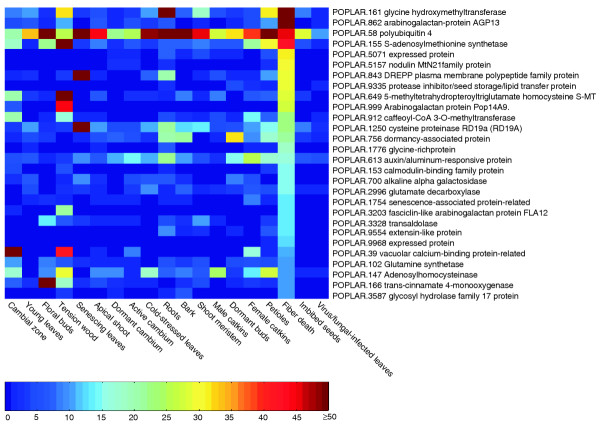
The most abundant transcripts in the fiber death library. The EST distribution in the different cDNA libraries of the POPULUSDB is shown for the 28 most abundant transcripts of the fiber death library. The transcripts are shown in descending order of EST abundance in the library. For a detailed description of the cDNA libraries, see [13]. The color code at the bottom of the figure indicates the number of ESTs in each library for each transcript.

A high abundance of a transcript suggests that the corresponding protein participates in a process that is important for the cell or tissue. We searched for highly abundant transcripts in the process of fiber-cell death by identifying in the fiber death library the POPULUSDB unigene clusters that had the highest numbers of ESTs. The 28 most abundant transcripts, shown in Figure 3, were also enriched in the fiber death library. Assuming a random distribution, the expected EST frequency in the fiber death library is 4.8% (4,867 ESTs in the fiber death library out of the total number of 102,019 ESTs in the POPULUSDB). All of the transcripts shown in Figure 3 displayed a higher frequency than that. In fact, all clusters except POPLAR.147, POPLAR.58, POPLAR.39, POPLAR.166 and POPLAR.613 had an EST frequency between 10-91% in the fiber death library.

The most abundant transcript in the fiber death library was glycine hydroxymethyltransferase (GHMT; POPLAR.161). GHMT was also highly abundant in several other libraries derived from xylem-containing tissues, such as the tension wood and the roots (Figure 3). GHMT has also been identified as one of the most abundant proteins in *Populus *xylem [15], *Arabidopsis *roots [16] and loblolly pine xylem [17]. GHMT is a key enzyme in one-carbon metabolism, catalyzing reversible conversion between serine and glycine to produce 5,10-methylenetetrahydrofolate, which can be used to recover methionine from 5-methyl-tetrahydrofolate and homocysteine [15]. One-carbon metabolism is known to be active in photorespiration, but its preferential expression in the late maturing fibers suggests that the *Populus *GHMT is also involved in some other process(es). Also, three other enzymes that participate in one-carbon metabolism were all highly abundant in the fiber death library. 5-Methyltetrahydropteroyltriglutamate-homocysteine *S*-methyltransferase (POPLAR.649) catalyzes biosynthesis of methionine, while *S*-adenosylmethionine synthetase (POPLAR.155) and adenosylhomocysteinase (POPLAR.147) are involved in methionine catabolism (Figure 3). It is possible that one-carbon metabolism is required during xylem maturation for the production of glycine, which is abundant in the cell wall proteins. *S*-adenosylmethionine, which is synthesized from methionine, can also be used for methylation reactions which occur during secondary wall formation. It is, however, unlikely that these enzymes are only needed for fiber-cell death, because they are also highly abundant in libraries derived from the cambial zone and tension wood (Figure 3).

Cell-wall proteins were highly abundant in the fiber death library (Figure 3). Arabinogalactan proteins (AGP) belong to a superfamily of proteoglycans encompassing several subclasses, such as classical AGPs and fasciclin-like AGPs. Both classical AGPs (POPLAR.862 and POPLAR.999) and a fasciclin-like AGP (POPLAR.3203) were highly abundant in the fiber death library (Figure 3). AGPs are a major proteinaceous constituent of the cell walls, but their function has remained unclear. A recent report on a hybrid protein containing AGP domains supports the hypothesis that AGPs have a critical function in mediating cell-cell interactions during vascular differentiation [18]. The fasciclin-like AGPs, on the other hand, seem to be involved in the control of cell adhesion [19,20]. AGPs have also been implicated in PCD [21,22]. AGP genes in clusters POPLAR.999 and POPLAR.3203 were also highly expressed in other libraries, especially the library from the tension wood-forming xylem tissues, suggesting that they have a more general function during cell-wall formation (Figure 3). However, the POPLAR.862 AGP was enriched in the fiber death library. POPLAR.862 has also been shown to be suppressed in a microarray analysis of tension wood where fiber-cell death is inhibited (S. Andersson-Gunnerås, E. Mellerowicz and B. Sundberg, personal communication), strongly indicating a role for this AGP in the stimulation of fiber-cell death. Two other cell-wall proteins, a glycine-rich protein (POPLAR.1776) and an extensin-like protein (POPLAR.9554), were also highly abundant and overrepresented in the fiber death library (Figure 3), suggesting that these proteins also have functions during the late maturation of xylem fibers.

**Figure 4:**
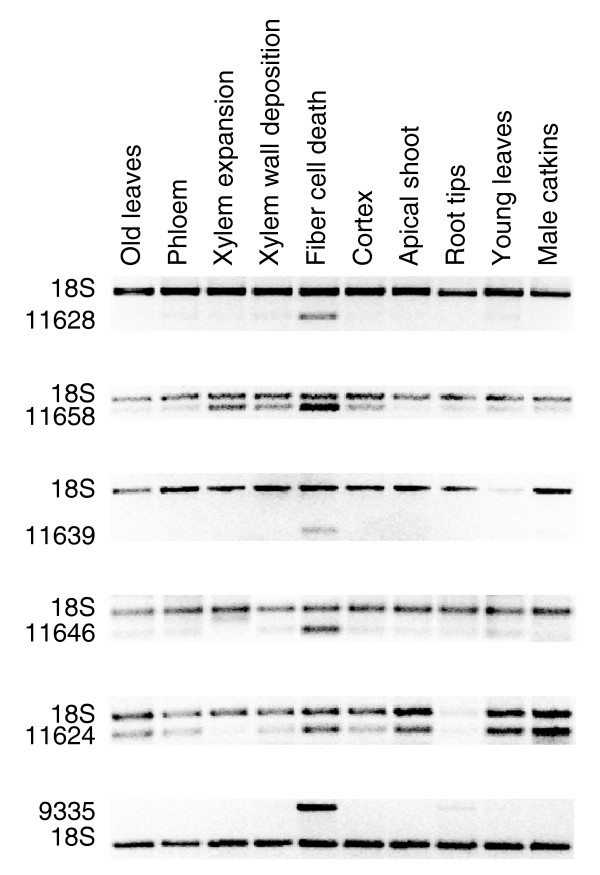
RT-PCR analysis of gene expression in different parts of a *Populus *tree. Expression of glycosyl hydrolase family 1 protein (POPLAR.11628), proline-rich protein (POPLAR.11658), oligopeptide transporter (POPLAR.11639), an expressed protein (POPLAR.11646), an F-box protein (POPLAR.11624) and a protease inhibitor/seed storage/lipid transfer protein (POPLAR.9335) is shown in relation to 18S DNA expression. Sampling of the different tissue types is described in Materials and methods.

A cysteine protease (POPLAR.1250) and a polyubiquitin (POPLAR.58) were highly abundant in the fiber death library (Figure 3), as well as in other libraries derived from tissues in which large proportions of cells are dying, such as senescing leaves, the root tissues and petioles. Cysteine proteases are believed to participate in the *post mortem *events of xylem elements [1]. The high abundance of polyubiquitin suggests that the ubiquitin-proteosome pathway participates in proteolytic events of xylem cells as well. A further transcript related to proteolysis and cell death was POPLAR.9335, which was highly abundant and also highly enriched in the fiber death library (Figure 3). It encodes a protein with an unknown function, but contains a domain found in lipid-transfer proteins, seed storage proteins and protease inhibitors. A similar kind of protein was earlier shown to regulate programmed cell death and plant defense [23]. The expression pattern of POPLAR.9335 was also analyzed by RT-PCR, and the results confirmed the specificity of this transcript in the xylem fibers undergoing cell death (Figure 4).

### The fiber death library-specific transcripts are putatively novel regulators of cell death

Analysis of the most abundant transcripts in the fiber-cell death library yielded a list of candidate genes with high expression levels. In order to identify fiber-cell death specific transcripts with lower expression levels we identified in POPULUSDB the clusters and singletons (non-clustered transcripts) that were unique to the fiber death library and not present in any of the other 18 EST libraries. In total, 71 clusters and 929 singletons were identified that were unique to the fiber death library (Additional data file 1). Singletons represent either rare transcripts or poorly sequenced regions of transcripts, and are therefore not necessarily indicative of fiber-death specific expression. Of the 71 fiber death library specific clusters, the 12 clusters having the highest abundance of ESTs are shown in Table 1. First, a microarray experiment was performed to confirm the expression pattern of these transcripts. Two samples were collected from the woody tissues of the stem; one (A) containing the zones where xylem fibers were in the process of cell expansion and secondary cell wall formation, and one (B) containing tissues where the fibers were undergoing cell death (see Figure 1). The sample from the fiber-cell death zone corresponded closely to the tissues collected for construction of the fiber death library, and should therefore show high expression of the transcripts unique to this library. The microarray analysis showed that, with the exception of POPLAR.11648, all seven fiber death library-specific clusters that were represented by more than three ESTs were also more highly expressed in the fiber-cell death sample compared to the early developing fibers (Table 1). Fiber death library-specific clusters with EST abundances below four showed varying results in the microarray analysis (Additional data file 1).

None of the transcripts that were shown to be unique to the fiber death library has previously been implicated in the regulation of cell death. The oligopeptide transporter (POPLAR.11639) is involved in amino-acid metabolism related to remobilization of nutrients from dying tissues, but the reason for the specific expression pattern of the other transcripts is not clear. Several of them seem to be membrane proteins or targeted to the endomembrane system, as predicted on the basis of their closest *Arabidopsis *homologs (Table 1). A glycosyl hydrolase of family 1 (POPLAR.11628) is the most abundant unique transcript in the fiber death library, but the substrates of this glucosidase and its exact role in fiber-cell death remain to be elucidated. Interestingly, the closest homologs of this protein are cyanogenic in nature, and it is tempting to speculate that cyanide production by this enzyme could participate in regulation of cell death in xylem fibers. Reverse transcription PCR (RT-PCR) analysis of five unique transcripts confirmed that POPLAR.11628 (nine ESTs), POPLAR.11639 (five ESTs) and POPLAR.11646 (four ESTs) were indeed very specifically expressed in xylem fibers undergoing cell death (Figure 4). POPLAR.11658 (six ESTs) and POPLAR.11624 (two ESTs) showed highest expression in the dying xylem fibers, but were also expressed in other types of *Populus *tree tissues (Figure 4).

### Combination of the *in silico *analysis with a microarray analysis refines selection of candidate regulatory genes

**Figure 5:**
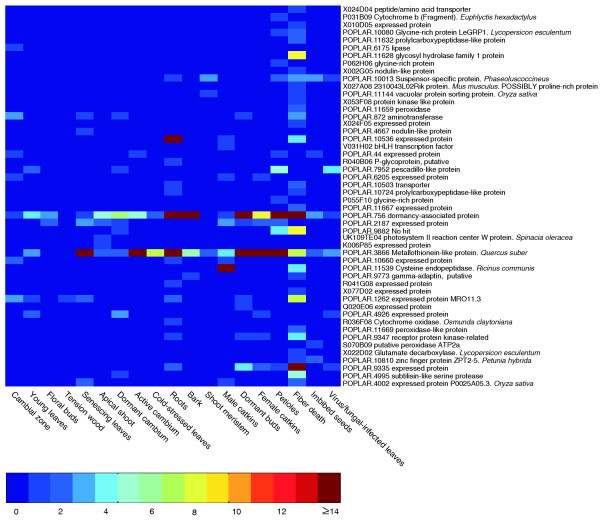
Gene expression in the 25K *Populus *cDNA array and in the POPULUSDB. The 50 most highly expressed transcripts in the microarray analysis of xylem fibers undergoing cell death are shown together with the corresponding library distribution in POPULUSDB. The transcripts are listed in descending order of expression ratio between the fiber-cell death sample and the early fiber development sample (see Figure 1). The color code at the bottom of the figure indicates the number of ESTs in each library for each transcript. The whole list is given in Additional data file 2.

The microarray analysis revealed both singletons and clusters that were represented only by two or three ESTs in the fiber death library, but were highly upregulated in the xylem fibers undergoing cell death (Additional data file 1). To combine the power of the POPULUSDB and the microarray analysis, transcripts were identified that had a high expression level in the dying fibers on the basis of the microarray analysis and that were enriched or unique in the fiber death library within the POPULUSDB. Figure 5 shows the 50 transcripts that were most upregulated in the dying xylem fibers compared to the early developing xylem on the basis of the microarray analysis. The most upregulated transcripts were generally highly enriched or unique to the fiber death library (Figure 5). Of the 20 most upregulated transcripts, nine were unique to the fiber death library and only five were completely absent from it (Additional data file 2). The good correlations between EST abundances in the fiber death library and gene expression in the microarray analysis verify the usefulness of the POPULUSDB in transcript profiling of xylem fiber death.

Among the most upregulated transcripts that were unique or highly enriched in the fiber death library, there are transcripts that participate in amino-acid metabolism and transport (X024D04, POPLAR.872), proteolysis (POPLAR.11632, POPLAR.4995, POPLAR.10724, X077D02), and also transcripts, such as kinases (X053F08, POPLAR.9347), nodulin-like proteins (X002G05, POPLAR.4667) and a CACTIN-like protein (POPLAR.11667), that seem to have signaling functions rather than mere cellular disintegration of the dying fibers (Figure 5). High expression of nodulin-like proteins in dying xylem fibers suggests that nodulins, which regulate nodule formation in response to *Rhizobium *infection, also have an important function in the maturation of xylem fibers. Interestingly, certain nodulins have been shown to regulate accumulation of reactive oxygen species (ROS) [24,25], and it is possible that nodulin-like proteins regulate ROS accumulation that occurs during the late maturation or cell death of xylem fibers. ROS accumulation is also implied by the high expression levels of peroxidases (POPLAR.11659 and 11669) and a protein kinase (singleton X053F08) that seems to encode the *Populus *ortholog of *Arabidopsis thaliana OPEN STOMATA 1 *(*OST1*; Figure 5). OST1 regulates abscisic acid (ABA)-mediated accumulation of ROS related to stomatal closure in *Arabidopsis*, and it has also been shown to be expressed in vascular tissues of *Arabidopsis *leaves and roots [26]. Other important proteins in the early signaling of fiber-cell death could include the basic helix-loop-helix transcription factor V031H02 and the C2H2 class zinc-finger protein POPLAR.10810. Interestingly, POPLAR.11144 is most similar to the *Arabidopsis *gene *VACUOLELESS1*, which is required for proper vacuole formation and autophagy [27]. In the *Z. elegans *cell culture system, the permeability and integrity of the vacuolar membrane regulates initiation of xylem-cell death [1], and our results suggest that *VACUOLELESS1 *could be involved in this regulation during the cell death of fibers.

Combining the *in silico *expression analysis in 19 different tissue types with a focused microarray analysis is expected to facilitate the identification of candidate genes better than either method alone. While the microarray analysis allowed selection of genes with high expression levels in xylem fibers undergoing cell death compared to early developing fibers, the *in silico *analysis of POPULUSDB facilitated exclusion of those genes that were highly abundant in other types of *Populus *tree tissues. The candidate genes selected here are potential novel regulators of cell death in xylem fibers.

### Comparison of proteases in three different cell death processes

The expression of serine, cysteine and aspartic proteases was analyzed in detail in the fiber death library, because proteases have well established roles in the control of cell death in both animal cells and plants [28,29]. We also compared their expression in the fiber death library and three other cDNA libraries: the leaf senescence library (library I) and the virus/fungal infected leaf library (library Y), which are expected to be enriched in transcripts related to cell death, and the tension wood library (library G), which should theoretically be devoid of fiber-death-related transcripts.

Cysteine proteases (CP) have been identified in xylem elements undergoing cell death [6,30]. Accordingly, inhibitors of cysteine proteases have been shown to impair maturation of xylem elements [31]. Cysteine proteases were highly abundant in the POPULUSDB, and they were usually not restricted to any of the cell death libraries X, I or Y (Table 2). However, the cysteine protease POPLAR.3310 was somewhat enriched in the fiber death library and not represented in the leaf senescence library. This transcript is most similar to *Arabidopsis XYLEM CYSTEINE PEPTIDASE 2 *(*XCP2*), which has been shown to be specifically expressed in xylem vessels of leaves and roots [32]. Interestingly, POPLAR.3310 was also present in the virus/fungal infected leaf library, suggesting that this CP is also activated during pathogen-induced cell death. In addition, a *VACUOLAR PROCESSING ENZYME *(VPE; POPLAR.3027), was enriched in the fiber death library (Table 2). Two *Arabidopsis *VPE isozymes, α-VPE and γ-VPE, have been shown to be activated during senescence, but only α-VPE was expressed in the vascular tissues [33]. In tobacco, VPE has been shown to regulate the integrity of the vacuolar membranes and pathogen-induced hypersensitive cell death [34]. Our data suggest that, in addition to hypersensitive cell death, VPE controls developmental cell death in xylem fibers, possibly through regulation of vacuolar integrity.

In contrast to the cysteine proteases, the serine proteases displayed higher specificity to given cell death processes (Table 2). This conforms well to the reported role of serine proteases in the early signaling of apoptotic cell death in virus-infected animal cells [29]. In plant cells, serine proteases have been implicated in pathogen-induced cell death [35,36], and serine protease activities have also been demonstrated during xylem-cell death [5,6]. Groover and Jones [4] showed that serine protease activity stimulated, and was necessary for, the death of *Z. elegans *tracheary elements grown *in vitro*. In accordance with these findings, a 40 kDa serine protease was shown to accumulate in the culture medium of *in vitro *tracheary elements [4]. Our data revealed the identities of two serine proteases possibly related to the regulation of xylem-cell death. The serine proteases POPLAR.10138 and POPLAR.4995 were enriched in the fiber death library, and also highly upregulated in the microarray analysis of the xylem fibers undergoing cell death (Table 2). A PSORT analysis [37] of protein sequences derived from ESTs and manually assembled genomic sequences supported extracellular locations for both POPLAR.10138 and POPLAR.4995 (data not shown).

The targets of these serine proteases are not known. Groover and Jones [4] suggested that the extracellular 40 kDa serine protease was responsible for activation of Ca^2+ ^channels, which is a prerequisite of xylem-cell death. Other possible targets are membrane-bound leucine-rich-repeat-containing proteins, which have been shown to interact with serine proteases during hypersensitive cell death [38]. One such target could be a plasma-membrane localized leucine-rich-repeat-containing receptor kinase that was unique to the fiber death library (singleton X021F12) and significantly upregulated during fiber death (PU27994; Additional data file 2). However, regardless of their targets, it seems evident from our data that modification of the extracellular matrix occurs during fiber-cell death by two extracellular serine proteases, probably as part of the early signal transduction process.

An aspartic protease has been localized specifically in the tracheary elements undergoing cell death [39]. In this analysis, we found no evidence for any fiber-death specific aspartic proteases. All four aspartic proteases identified in POPULUSDB were either present in several cell death libraries or also in the tension wood library, where cell-death-related transcripts should not accumulate (Table 2).

### Hormonal control of fiber maturation

*Arabidopsis *has been used to analyze transcriptomes during xylem development [40,41]. In *Arabidopsis*, formation of the vascular cambium, giving rise to the so-called secondary growth, takes place in the hypocotyls after two to three months of growth requiring continuous removal of the inflorescence stems [42]. The early development and maturation of xylem vessels and fibers during the secondary growth of the hypocotyl is similar to *Populus *except for that the fibers in *Arabidopsis *do not die in the highly coordinated manner as in *Populus*, even after an extended growth period of three months (our unpublished work). It is therefore possible to analyze similarities between *Populus *and *Arabidopsis *in the process of fiber maturation but not cell death.

To identify common patterns in the transcriptomes of *Populus *and *Arabidopsis *during fiber maturation, we identified *Arabidopsis *homologs to the *Populus *genes that were upregulated at least two times (*P *< 0.001 and *B *> 0) in the fiber cell death sample (late maturing fibers; sample B) compared to the early fiber development (sample A). This dataset, denoted as '*Populus *B/A', was compared to two previously published *Arabidopsis *datasets [41]. The first *Arabidopsis *datasets, denoted as 'treatment', consists of genes that were upregulated at least two times during secondary growth (9 weeks of growth) compared to the primary growth of the hypocotyls (3 weeks of growth), and is therefore expected to enrich transcripts related to secondary growth including fiber maturation. The second *Arabidopsis *dataset, denoted as 'xylem', consists of genes that were upregulated more than two times in secondary xylem tissues compared to bark tissues of hypocotyls, and is expected to enrich transcripts related to all aspects of xylem development during secondary growth including death of the xylem vessels and fiber maturation. The common features of these datasets are shown in Additional data file 3. Cell-death-related transcripts were rarely shared by the different datasets due to the sampling method for the comparisons 'treatment' and 'xylem'. However, a large number of transcription factors and plant hormone-related transcripts were often shared by the *Arabidopsis *and the *Populus *datasets. We will discuss here the latter group of transcripts as very little previous knowledge exists on the role of plant hormones in the late maturation events of xylem fibers.

Indole-3-acetic acid (IAA) is an auxin-type plant hormone which regulates several different developmental processes in plants and also the activity of the vascular cambium and xylem formation [43]. In *Populus *stems, IAA shows a steep radial concentration gradient across the stem with the highest concentration close to the vascular cambium and a very low concentration in the late-maturing xylem [11]. Several transcriptional regulators of IAA-related gene expression, such as the auxin response factor (ARF) family proteins as well as the auxin/indoleacetic acid (IAA) family proteins, were observed in all the datasets (Additional data file 3), suggesting involvement of these proteins not only in the early development of secondary xylem, as suggested by the high concentration of IAA in these tissues, but also during late maturation of the xylem fibers. The role of IAA during late maturation of fibers is not clear, but it is possible that functional IAA signaling is required for the survival of the cells. At2g21620 encodes a universal stress protein (USP) family member, which is regulated by auxin [44]. USPs have been implicated in the regulation of stress-related metabolic shifts, and activation of the auxin-regulated USP during secondary growth in *Arabidopsis *and during late fiber maturation in *Populus *(Additional data file 3), suggests a role for IAA in late maturing xylem fibers that experience some kind of stress. This could be osmotic stress due to condensation of the cytoplasmic contents or nutrient depletion due to the increasing distance from the nutrient-transporting phloem cells. The role of IAA during cellular stress is supported also by the fact that IAA induces expression of enzymes involved in the biosynthesis of ethylene [45], which is a plant hormone that is produced in response to several different stress conditions.

Ethylene does not seem to be needed for normal xylem development on the basis of the fact that ethylene-insensitive genotypes in *Arabidopsis *and in other species grow normally. However, ethylene biosynthesis is activated in gravitationally stimulated *Populus *stems, that is, when the stem is displaced from its vertical position, resulting in the production of tension wood [46]. By analogy with several other cell-death processes in plants [47], it is expected that ethylene is also involved in regulation of xylem cell death. This is supported by the activation of several ethylene-related transcripts in both *Arabidopsis *and the late-maturing xylem fibers in *Populus *(Additional data file 3). *ETHYLENE-INSENSITIVE 3 *(*EIN3*), *EIN3-BINDING F-BOX 1 *(*EBF1*) and *ETHYLENE RESPONSE SENSOR 1 *(*ERS1*) mediate known parts of ethylene signal transduction [48]. *EBF1 *and *ERS1 *are also induced by ethylene. Because both *EBF1 *and *ERS1 *function to suppress ethylene signaling, it seems that the ethylene signal required for xylem maturation needs to be suppressed or only transiently activated.

Comparison of the datasets suggests involvement of two additional plant hormones. *PATHOGENESIS-RELATED PROTEIN *and *PHENYLALANINE AMMONIA-LYASE 2 *are related to salicylic acid (SA) signaling and biosynthesis, respectively, and *CORONATINE INSENSITIVE 1 *to jasmonic acid (JA) signaling. Both JA and SA control stress-related processes, such as cell death and other defense responses to pathogens [47]. The transcriptional activation of these genes in the *Arabidopsis *xylem tissues and in the late maturing *Populus *fibers suggests that both JA and SA are involved in the regulation of cell death also in xylem fibers (Additional data file 3).

## Conclusions

Even though *Arabidopsis *is a suitable model system for studying early vascular development and primary growth, it cannot be easily used for studying secondary growth of the stem. Xylem fibers are formed in *Arabidopsis *only after two to three months of growth, which is equivalent to the time required to grow *Populus *trees to a size that allows collection of large amounts of woody tissues from the stem. In addition, because of the larger diameter growth of the tree stem, tissues can be easily collected from the various developmental phases without mixing the different tissue types [43]. Therefore, *Populus *has several advantages over *Arabidopsis *for wood analysis. Development of appropriate genomic and bioinformatic tools has further strengthened *Populus *as the main model system for wood formation.

This analysis fully exploited the advantages of the *Populus *system. Combining *in silico *analysis of the POPULUSDB with a microarray analysis using the novel 25K *Populus *microarrays revealed a number of candidate genes that were unique and highly abundant in the late-developing woody tissues, and possibly related to the regulation of cell death in xylem fibers. We found fiber death-specific transcription factors, as well as nodulins and subtilisin-like serine proteases, all of which are expected to play a role in the early signaling of fiber-cell death. Sequences corresponding to the *Populus *homolog of *Arabidopsis OPEN STOMATA 1 *and peroxidases suggest involvement of ROS and ABA in fiber-cell death signaling as well. Specific expression patterns of downstream components, such as the *Populus *homolog of *Arabidopsis XYLEM CYSTEINE PEPTIDASE 2 *and the oligopeptide transporters, support the importance of these proteins in protein degradation and remobilization of nutrients in the dying fibers. Interestingly, two proteins that are known to regulate vacuolar assembly and vacuole integrity in other cellular processes, the *Populus *homolog of *Arabidopsis VACUOLELESS1 *and a *VACUOLAR PROCESSING ENZYME*, were specifically expressed during fiber-cell death. Permeability of the vacuolar membrane and vacuolar integrity are known to regulate death of the xylem cells, and we can now for the first time suggest candidate regulatory proteins for this process. Taken together, our analyses have identified a number of candidate genes that may be important in the regulation of fiber-cell death. Overexpression and transcriptional suppression experiments should be undertaken to elucidate the function of these genes in the process of fiber-cell death and their impact on economically important traits of woody tissues.

## Materials and methods

### Computational biology and database analyses

Construction of the fiber death library and the POPULUSDB [14] is described in [13]. Data were analyzed using mysql, PHP, C++ and Filemaker software. The gene ontology (GO) comparison was made by listing all GO terms [14] in the hierarchy for each clone and selecting clones from appropriate GO hierarchy levels. Around 10% of the clones were assigned to more than one class. The EST clone distribution within POPULUSDB was performed using mysql and Filemaker, and visualized with the R software package [49] according to the program code described in [13]. Annotations were derived from BLASTX analysis against the *Arabidopsis *proteome or the Swiss-Prot database in cases where no *Arabidopsis *proteins with sufficient homology were identified according to an annotation pipeline described in [50].

### Preparation of the 25K *Populus *cDNA microarray

The microarrays used here constitute the second generation of the global *Populus *cDNA microarrays and contain in total 24,735 different cDNA fragments. This array is based on the first generation 13K *Populus *array [51] with clones from seven cDNA libraries, representing: the cambial zone (AB), young leaves (C), floral buds (F), tension wood (G), senescing leaves (I), dormant cambium (UA), and active cambium (UB). The 25K array contains clones from 12 additional cDNA libraries, representing the apical shoot (K), cold-stressed leaves (L), roots (R), bark (N), shoot meristem (T), male catkins (V), dormant buds (Q), female catkins (M), petioles (P), fiber death (X), imbibed seeds (S) and virus/fungal infected leaves (Y). For a detailed description of the construction and sequencing of the cDNA libraries, see [13]. All clones in the unigene set were resequenced from both the 5'-end, as were the original EST sequences, and the 3'-end. Each unigene clone is defined by a PU number on the microarray. Sequence information of the clones can be found in the POPULUSDB [14].

Plasmid preparations were made in a 96-plate format from bacterial cell suspensions with a Montage 96 Plasmid Prep kit (Millipore) using a Biorobot 8000 molecular biology workstation (Qiagen). PCR amplifications were done in 100 μl reaction volumes, and purified in Montage PCR_384 _Filter Plates (Millipore) with a Biorobot 8000 workstation (Qiagen). The purified PCR products were dissolved in 40 μl of 30% DMSO and split between a storage plate and a printing plate.

The microarrays were printed with a QArray (Genetix) instrument with 24 SMP2.5 pins (Telechem) on Ultra GAPS slides (Corning). The 24,735 cDNA fragments, together with eight copies each of the 23 different Lucidea Universal Scorecard controls (Amersham Biosciences), were spotted with a feature center-to-center distance of 180 μm. The quality of the spotted slides was assessed by staining with Syto61 (Molecular Probes) and by hybridization with random nonamers. The slides were UV cross-linked at 250 mJ/cm^2 ^followed by baking at 75°C for 2 h.

### Microarray analysis

Samples for RNA isolation were collected from the base of the stem of a 6-month-old hybrid aspen tree (*Populus tremula x tremuloides*) grown in a greenhouse. The bark was peeled off, and the developmental phases of the xylem were identified by light microscopy and by the texture and color of the different tissue types (Figure 1). The A sample, consisting of the remains of the cambial zone, expanding xylem and secondary cell wall depositing xylem, was collected by scraping the part of the xylem that was yellowish in color and still relatively soft with a knife. The B sample, consisting of the thick-cell-walled and late maturing xylem fibers approaching cell death, was light yellow in color and relatively difficult to scrape with the knife. The B sample was scraped to the border of the dead wood, which was discernible by its completely white color, dryness and hard texture. Total RNA was prepared from the two samples according to [52], and mRNA was prepared from 1 μg total RNA using paramagnetic oligo(dT) beads (Dynabeads, Dynal Biotech) in a 10 μl elution volume.

For cDNA synthesis, the mRNA sample was sheared by repeated suction into a 10 μl syringe with a beveled, non-coring needle point (Hamilton Bonaduz AG). First-strand cDNA was prepared with 1 μg oligo-dT_15 _primer and 200 U SuperScript II RNase H^- ^reverse transcriptase (Invitrogen). Second-strand cDNA was prepared in a final volume of 150 μl at 16°C for 2 h with 10 U *Escherichia coli *DNA ligase (Invitrogen), 40 U *E. coli *DNA polymerase I (MBI Fermentas), 2 U RNase H (USB, GE Healthcare Bio-Sciences) in a buffer containing 0.2 mM of each dNTP, 19 mM Tris-HCl (pH 6.9), 91 mM KCl, 4.5 mM MgCl_2 _and 10 mM (NH_4_)_2_SO_4_. A further incubation was performed at 16°C for 20 min with 2 U T4 DNA polymerase (Invitrogen). The reaction was stopped with 10 μl 0.5 M EDTA, and the cDNA was recovered by phenol and chloroform:isoamylalcohol extraction followed by ethanol precipitation. The precipitates were dissolved in 2 mM Tris-HCl (pH 7.5) and purified using Autoseq G-50 columns (Amersham Biosciences). A blunt-end adapter was created by annealing two single-stranded oligonucleotides, (0.5 mmol MarAdLong: 5'-CTAATACGACTCACTATAGGGCTCGAGCGGCCGCCCGGGCAGGT-3' and 0.5 mmol MarAdShort 5'-PO_4_- ACCTGCCC- NH_4_-3') in a ligase buffer containing 66 mM Tris-HCl (pH 7.6), 5 mM MgCl_2_, 5 mM DTT and 7.5 μg BSA in a total volume of 15 μl with a 0.7°C/min temperature gradient from 50°C to 10°C. The adapter was ligated to the 5' template strand with 10 U T4 DNA ligase (Invitrogen) in a ligase buffer containing 33.8 mM Tris-HCl (pH 7.6), 2.56 mM MgCl_2_, 2.56 mM DTT and 24 μg BSA in a total volume of 48.8 μl at room temperature. The cDNA was purified with a QIAquick PCR purification kit (Qiagen).

The cDNA was amplified by PCR in a 100-μl volume containing 0.2 mM of each dNTP, 0.75 μM MaraAP1 (5'-CCATCCTAATACGACTCACTATAGGGC-3'), 0.75 μM oligo-dT_15_, 67 mM Tris-HCl (pH 8.8), 4 mM MgCl_2_, 16 mM (NH_4_)_2_SO_4_, 3 μg BSA and 0.6 μl AmpliTaq DNA polymerase (Applied Biosystems). The PCR procedure was 95°C for 1 min, 72°C for 5 min, addition of the AmpliTaq, and 17 to 29 cycles of 95°C for 1 min, 50°C for 1 min and 72°C for 2 min. The appropriate cycle number was defined as two cycles before saturation of the PCR product as detected by gel electrophoresis. The PCR product was purified using a QIAquick PCR purification kit and its concentration was measured by spectrophotometry (NanoDrop ND-1000, NanoDrop Technologies).

Labeling of the amplified cDNA samples was performed by direct incorporation of 3 μl Cy3-dUTP or Cy5-dUTP (Amersham Biosciences) in an asymmetric PCR reaction with 100 ng cDNA, 1 μM MaraAP1 primer, 0.6 μl AmpliTaq, 67 mM Tris-HCl (pH 8.8), 4 mM MgCl_2_, 16 mM (NH_4_)_2_SO_4_, 80 μM of each dATP, dCTP, dGTP and 20 μM dTTP in a total reaction volume of 50 μl. The PCR conditions were 95°C for 1 min followed by nine cycles of 95°C for 30 sec, 50°C for 30 sec and 72°C for 10 min. The PCR product was purified with a QIAquick PCR purification kit and eluted twice in 30 μl 4 mM KPO_4 _buffer (pH 8.5). The final volume was decreased to 41 μl.

Microarray hybridizations, as well as scanning of the slides and image analysis were done according to [53]. The two samples A and B were hybridized against each other five times (including dye-swaps). The microarray raw data, including tiff and gpr files from scanning and image analysis, is deposited to the UPSC-BASE microarray database [54]. For statistics, *B-*values based on Bayesian statistics [55] and parametric *t*-tests were obtained with the program R, version 1.8.1 [49]. The microarray data shown in Additional data files 1 and 2 includes the log_2 _differential expression ratio (*M*) between the two samples B and A, *P*-value from the *t*-test and the *B*-value.

### RT-PCR analysis

Samples were collected from ten different tissue types of a 6-month-old hybrid aspen (*Populus tremula *× *tremuloides*) tree grown in the greenhouse. Stem tissues; cortex, phloem, expanding xylem, secondary cell wall-depositing xylem, and fiber-cell death tissues, were collected by scraping with a knife from the base of the stem (see Figure 1). The developmental phase of the different xylem tissues was verified in transverse sections of the stem. The xylem fiber-cell death sample corresponded to the tissues collected for the B sample in the microarray analysis (see above) and the tissues prepared for construction of the fiber death cDNA library analyzed in this study (for a description of the library see [13]). Other tissues collected for the RT-PCR analysis were the apical shoot, root tips, young leaves, old leaves and male catkins.

RNA was prepared according to [52]. The samples obtained were treated with RQ1 DNase (Promega), and cDNA was produced using SuperScript II RNase H- (Invitrogen) and random decamers (Ambion), followed by RNase H treatment. Two microliters of cDNA was PCR-amplified using gene-specific primers and an appropriate mixture of the universal 18S gene-specific primers and the 18S competimers (Ambion). The gene-specific primers were ACAGATGAAGCGTTCAGCAA and CAGTGCAGCACACCTAGGAA for cluster POPLAR.11628, CAGGAAAGCTCTCCGTTTCTT and TCAAAGCTCTTCCCTTCTGC for POPLAR.11658, AATCCCATGAATATTACCCCTAGA and TCTCTTGCATGGGTAGACATTTT for POPLAR.11639, ACCTCCATAGCCACCCAAG and CTGCAAGCTGATGCAGAAGT for POPLAR.11646, TGAACAGCAGGAGGTGTGAG and AACAGGTGTCCCCATCTGAG for POPLAR.11624, and TGGCAACTCCAATGAAGAAC and CACCAACAGTTTATTTATTATTCAGATG for POPLAR.9335.

### Analysis of proteases in the POPULUSDB

A list of *Populus *proteases was compiled, based on known protease sequences of *A. thaliana *collected from the following databases: the *Arabidopsis *Information Resource [56], TrEMBL [57], Merops [58], InterPro [59], TIGR [60] and Munich Information Center for Protein Sequences [61]. Sequences of 602 *Arabidopsis *proteases were found. The contig sequences of the POPULUSDB were then queried with BLASTX against the *Arabidopsis *proteases. Only the *Populus *sequences that obtained a BLASTX score higher than 100 and were annotated as proteases or as coding for proteins with a biological function related to proteolysis or peptidolysis in the TIGR database were accepted. The corresponding clusters, having three or more ESTs in any of the libraries of interest (tension wood, senescing leaves, fiber death and virus/fungal infected leaves) in POPULUSDB were chosen for the analysis.

## Additional data files

The following additional data is available with the online version of this article. Additional data file 1 is a table containing the complete list of fiber death library-specific transcripts and the corresponding gene expression data from the microarray analysis. Additional data file 2 is a table containing the complete gene expression data in xylem fibers undergoing cell death using the 25K *Populus *cDNA array and the POPULUSDB. Additional data file 3 is a table containing a comparison of the microarray data from xylem tissues of *Populus *and *Arabidopsis*. Additional data file 4 is a table showing GenBank accession(s) for the EST sequences(s) in each POPULUSDB unigene cluster and singleton.

## Supplementary Material

Additional File 1 The complete list of fiber death library-specific transcripts and the corresponding gene expression data from the microarray analysis. The list shows clusters and singletons unique to the fiber death library within the POPULUSDB, the number of EST sequences in each cluster, annotation, closest *Arabidopsis *gene and the BLASTX value (*E*), and the corresponding gene expression from the microarray analysis. The log_2 _expression ratio (*M*) is calculated between gene expression in sample B (fiber-cell death) and sample A (early fiber development). A statistically significant difference in gene expression is predicted for transcripts having *P*<0.001 (*t*-test) and *B>*0 (Bayesian statistics [55]). Click here for file

Additional File 2 The complete gene expression data in xylem fibers undergoing cell death using the 25k *Populus *cDNA array and the POPULUSDB. Transcripts and the corresponding library distribution in POPULUSDB are listed in descending order of expression ratio from the microarray analysis. The log_2 _expression ratio (*M*) is calculated between gene expression in sample B (fiber-cell death) and sample A (early fiber development). A statistically significant difference in gene expression is predicted for transcripts having *P*<0.001 (*t*-test) and *B>*0 (Bayesian statistics [55]). The annotations and the *E *values were derived from BLASTX analysis against the *Arabidopsis *proteome or the Swiss-Prot database according to an annotation pipeline described in [50]. The PU numbers define the unigene clone numbers on the array. Click here for file

Additional File 3 Comparison of the microarray data from xylem tissues of *Populus *and *Arabidopsis*. The *Populus *genes were selected that were statistically significantly more than two times upregulated in the fiber cell death sample (late maturing fibers; B) compared to the early fiber development (A). The automatically assigned *Arabidopsis *homologs [14] were listed for these *Populus *genes (column '*Populus *B/A'), and compared to *Arabidopsis *genes that were previously shown to be upregulated at least two times during secondary growth compared to primary growth of the inflorescence stems (column 'Treatment') or in xylem tissues compared to bark of the hypocotyls (column 'Xylem') [41]. The list shows the genes that were shared by the *Populus *dataset and at least one of the *Arabidopsis *datasets. '1' denotes the presence, and '0' the absence of a particular gene in a dataset. The annotations were derived from the *Arabidopsis *Information Resource (TAIR) 28/01/2005. The PU numbers, corresponding to the unigene clones of the selected *Populus *genes, are shown for each homologous *Arabidopsis *gene. Click here for file

Additional File 4 A table showing GenBank accession(s) for the EST sequences(s) in each POPULUSDB unigene cluster and singleton. A table showing GenBank accession(s) for the EST sequences(s) in each POPULUSDB unigene cluster and singleton. Click here for file

## Figures and Tables

**Table 1:** The unique transcripts in the fiber death library within the POPULUSDB

Cluster	Transcript	Number of ESTs	Most similar *Arabidopsis *gene	*E*	Cellular compartment	Expression ratio B/A	*P*	*B*
11628	Glycosyl hydrolase family 1 protein	9	At3g60130	4.2E-126	Mitochondrion	29.0	0.00000060	11.8
11658	Proline-rich protein family	6	At2g27390	1.6E-18	Membrane	2.5	0.00015	3.1
11639	Oligopeptide transport family protein	5	At2g37900	1.3E-05	Membrane	2.8	0.00076	1.5
11646	Expressed protein	4	At2g22080	6.1E-01	Endomembrane system	7.9	0.0000091	7.2
11619	Expressed protein	4	At3g45160	2.8E-10	Endomembrane system	4.8	0.0000061	7.8
11625	Transferase family protein	4	At2g25150	3.8E-90		1.7	0.00036	1.9
11648	Basic helix-loop-helix family protein	4	At1g61660	2.9E-54	Chloroplast	1.3	0.14	-5.5
11665	Peroxidase	3	At1g05260	5.3E-119	Endoplasmic reticulum	5.5	0.0000079	7.4
11640	Expressed protein	3	At3g19340	1.2E-43	Membrane	1.1	0.83	-7.7
11662	Expressed protein	3	-	-	-	0.8	0.30	-6.1
11630	PHD-type zinc finger protein	3	At2g36720	1.2E-29	Nucleus	0.7	0.0014	0.2
11653	Ubiquinone reductase	3	At1g76340	9.9E-01	Membrane	0.5	0.010	-1.9

The table shows clusters that are unique to the fiber death library and have an EST abundance above two. The number of EST sequences in each cluster, the most similar *Arabidopsis *gene and the BLASTX value (*E*), and the corresponding gene expression from the microarray analysis are shown. The expression ratio is calculated between gene expression in sample B (fiber-cell death) and sample A (early fiber development). The transcripts were each represented on the microarray by a single cDNA clone. A statistically significant difference in gene expression is predicted for transcripts having *P *< 0.001 (*t*-test) and *B *> 0 (Bayesian statistics [55]). The whole list can be seen in Additional data file 1.

**Table 2:** Expression of cysteine, serine and aspartic proteases in POPULUSDB and in a microarray analysis

Protease	Cluster	Transcript	Most similar *Arabidopsis *gene	*E*	Number of ESTs in each library					Expression ratio B/A					
											
					I	X	Y	G	Tot						
Cysteine	11539	Cysteine endopeptidase *CysEP*	At5g50260	7.1e-146	0	4	0	0	30	*14.9					
	1250	Papain-like cysteine proteinase *RD19A*	At4g39090	1.4e-133	51	23	2	3	211	1.2	*3.0	1.1	*3.4	*4.1	1.4
	289	Papain-like cysteine proteinase	At5g43060	1.3e-103	16	1	1	1	32	0.9	1.4	1.4			
	3027	Vacuolar processing enzyme	At4g32940	9.2e-200	4	8	1	1	34	*3.0	*3.8	*3.1	*3.3		
	3310	Papain-like cysteine endopeptidase *XCP2*	At1g20850	1.7e-144	0	5	1	2	14	*0.7	*0.2				
	4102	Papain-like cysteine proteinase *SAG12*	At5g45890	8.8e-111	13	0	0	0	14	1.0	0.9				
	5498	Papain-like cysteine proteinase *AALP*	At5g60360	3.4e-129	4	1	0	0	18	1.3	*1.8	*2.3			
	7212	Papain-like cysteine proteinase *RD21A*	At1g47128	2.6e-169	28	5	2	3	59	2.2					
															
Serine	10138	Subtilisin-like serine protease	At1g01900	9.1e-107	0	3	0	0	5	*5.0					
	4702	Prolyl endopeptidase	At1g76140	1.6e-52	4	0	0	0	9	1.1	1.2				
	4905	Serine carboxypeptidase III-like protein	At3g10410	9.8e-194	0	4	0	2	16	1.6	1.3				
	4995	Subtilisin-like serine protease	At1g20160	7.0e-68	0	5	0	0	6	*12.2					
	8771	DegP protease	At3g27925	6.2e-60	9	0	0	0	9	1.6					
															
Aspartic	3648	Aspartic-type endopeptidase (phytepsin)	At1g11910	3.5e-208	6	3	0	1	26	0.9	*2.5				
	4286	Aspartic-type endopeptidase (family A1)	At5g43100	3.5e-41	3	1	0	0	4	1.0	1.2	1.2			
	541	Aspartic-type endopeptidase (family A1)	At2g03200	3.0e-124	0	6	0	10	19	1.1	0.7				
	915	Aspartic-type endopeptidase (phytepsin)	At1g11910	1.3e-105	3	3	1	1	35	1.2	1.0	1.3			

Distribution of the ESTs is shown in the cDNA libraries of senescing leaves (I), fiber death (X), virus/fungal infected leaves (Y) and tension wood (G) of the POPULUSDB. Tot, the total number of ESTs in the POPULUSDB. Note that the Y library was partially subtracted and only 1,395 ESTs were sequenced from this library. *E *represents BLASTX statistics for the most similar *Arabidopsis *gene. The expression ratio was calculated between gene expression in sample B (fiber-cell death) and sample A (early fiber development). Several transcripts were represented on the microarray by multiple cDNA clones. The asterisks denote statistically significant differences in gene expression when *P *< 0.001 and *B *> 0. The complete microarray data, including statistics, can be found in Additional data file 2.
